# Tapered Optical Fiber Sensor Coated with Single-Walled Carbon Nanotubes for Dye Sensing Application

**DOI:** 10.3390/mi14030579

**Published:** 2023-02-28

**Authors:** Aleksandr A. Polokhin, Yuri P. Shaman, Pavel A. Itrin, Ivan S. Panyaev, Artem A. Sysa, Sergey V. Selishchev, Evgeny P. Kitsyuk, Alexander A. Pavlov, Alexander Yu. Gerasimenko

**Affiliations:** 1 Institute of Biomedical Systems, National Research University of Electronic Technology MIET, Shokin Square 1, 124498 Moscow, Russia; 2 Scientific-Manufacturing Complex “Technological Centre”, Shokin Square 1, bld. 7 off. 7237, 124498 Moscow, Russia; 3 S.P. Kapitsa Research and Technology Institute, Ulyanovsk State University, 42 Leo Tolstoy Str., 432017 Ulyanovsk, Russia; 4 Institute of Nanotechnology of Microelectronics of the Russian Academy of Sciences, Leninsky Prospekt 32A, 119991 Moscow, Russia; 5 Institute for Bionic Technologies and Engineering, I.M. Sechenov First Moscow State Medical University, Bolshaya Pirogovskaya Street 2-4, 119991 Moscow, Russia

**Keywords:** carbon nanotubes, tapered optical fiber, dye sensing, functional coating

## Abstract

The present study aimed to improve the optical sensing performance of tapered optical fiber sensors toward aqueous Rhodamine B solution of different concentrations by applying single-walled carbon nanotubes (SWCNTs). The functional coating was formed on the surface of the tapered optical fiber sensor using an aerosol layer-by-layer deposition method. Before deposition, the SWCNTs were processed with multistage liquid-phase treatment in order to form a stable dispersion. The effect of SWCNT treatment was investigated through Raman spectroscopy. The deposition of 220 layers caused a reduction of up to 60% of the initial optical power of radiation propagating through the optical fiber core. The optical fiber sensor coated with SWCNTs demonstrated significantly higher sensitivity compared to a non-coated sensor in the range of 2–32 mg/L of Rhodamine B concentration in an aqueous solution. The experimental results demonstrated that the sensitivity was increased 10 times from 32 (mg/L)^−1^, for the non-coated sensor, up to 317 (mg/L)^−1^ after SWCNT coating deposition. Moreover, the SWCNT-coated sensor demonstrated high repeatability that allowed for the evaluation of the concentration regardless of the previously analyzed dye concentration.

## 1. Introduction

Nowadays, optical sensors are used to detect changes in different optical parameters, such as absorbance, reflectance, refractive index (RI), fluorescence, etc., which depend on the physical and chemical parameters (temperature, humidity, pressure, chemical composition, etc.) of the analyzed environment. Particularly, with the improvement of optical fiber manufacturing and processing technologies, sensors based on optical fiber are widely used [1]. There are two classes of optical fiber sensors: extrinsic (the change is outside the fiber) and intrinsic (the fiber is used as a transducer).

Due to the plasticity of SiO_2_ glass at high temperatures, one of the simplest and most frequently used types of optical fiber sensors is a tapered one. The tapered fiber technique allows for the measurement of parameters of the analyzed environment, such as chemical composition or RI, as the light propagating through the tapered optical fiber region interacts with the surrounding medium. The part of the tapered region with the smallest diameter is called the waist and is bound by conical sections with the unperturbed surrounding fiber.

The tapered optical fiber sensor properties mainly depend on the following parameters: the RI of the surrounding medium, the waist diameter, and the tapering angle (geometrical profile of conical sections) [2]. For example, the propagating light interaction with the surrounding medium increases with a decreasing difference between the RI of the fiber material and the surrounding medium and with the decreasing diameter of the taper waist [3].

Additionally, the properties of optical fiber sensors could be changed by functional coatings, such as polymers [4], nanoparticles [5], or nanostructures [6]. Carbon nanotubes (CNTs) are extremely attractive for application as a functional coating due to their unique structure and properties [7,8].

CNTs have excellent electrical [9,10] and optical [11] properties as well as high chemical stability [12] and mechanical strength [13,14]. Particularly, in the case of aqueous environment analysis, it is suitable to coat tapered optical fiber sensors with CNTs with high adhesion to the fiber surface [15].

An emphasis should be placed on the optical properties of CNTs [11]. Recent decades have seen the successful application of carbon nanotubes in the generation of ultrashort pulses within optical fiber lasers. CNT films, as saturation absorbers, have superior performance in inducing mode-locking and broadband operation [16]. The drawbacks of CNTs are due to nanotube agglomeration and large nanotube energy distribution that produces a high non-saturable loss in the laser cavity [17]. However, these issues are successfully solved by applying new technical approaches in saturation absorber development, including fused tapered fiber solutions [18].

Various industries generate large amounts of contaminated dye effluents. For example, contact with Rhodamine B dye, an organic chloride salt, could lead to skin dermatitis, allergic reaction, attention problems, hyperactivity, and restlessness [19]. In recent years, Rhodamine B has been one of the most common contaminants of the aquatic environment, particularly because of the textile industry [20]. Regarding that concern, Rhodamine B concentration should be monitored in order to prevent critical ecological and medical consequences. In the context of environmental monitoring, it is necessary to detect the concentration of pollution agents below the critical value to prevent irreversible consequences. For example, in the case of Rhodamine B, the lethal concentration of 10% (LC10) for crustaceans is 18 mg/L [21]; hence, the sensor should demonstrate low-level detection below this value. In this case, CNTs can improve the performance of the sensors being developed according to their unique interaction with chemical compounds [22].

In this study, a tapered optical fiber sensor coated with CNTs was designed to measure the concentration of Rhodamine B dye in an aqueous solution. In the experimental part of the study, the impact of a functional coating formed with carbon nanotubes was investigated. The spectral responses of the developed tapered optical fiber sensor toward different concentrations of Rhodamine B (2 mg/L to 32 mg/L) were also reported.

## 2. Materials and Methods

### 2.1. Tapered Fiber Sensor

A commercial, single-mode optical fiber, SMF-28e (Corning, New York, NY, USA), with core and cladding diameters of 8.2 µM and 125 µM, respectively, was applied to fabricate the tapered fiber sensor. A tapered section of the fiber was formed with a custom-designed workstation.

The tapering fiber workstation operated by pulling both ends of the optical fiber while its uncoated part was heated by a hydrogen flame torch operating at temperatures above 1000 °C. The tapering process was managed by controlling the torch flame, motion stages, pulling length, and continuously measuring the optical losses. As a result, we were able to produce silica tapered fibers with a diameter of 6 µM. At a wavelength of 550 nm, the standard attenuation of the fiber under test was approximately 10 dB/km. The presence of conical sections in the optical fiber increased attenuation. Thus, it was crucial to ensure uniform tapering and avoid surface roughness to minimize insertion losses [23]. The optical loss of the tapered fiber was 1.32 dB.

Considering the extremely small diameter of the taper waist, the mechanical strength of the sensitive part was very low. To prevent damage, the sensor was mounted in a quartz cell and fixed with epoxy glue right after the fiber-tapering process. The geometrical properties of the tapered section were investigated through optical microscopy BLM M-1 (LOMO, Petersburg, Russia).

### 2.2. Deposition of CNTs

In order to create stable, homogeneous dispersion in DFMA, the SWCNTs (SWCNT-80, Uglerod-ChG, Moscow, Russia) were soaked in HCl for 24 h and followed by treatment in H_2_O_2_ at the temperature of 100 °C for 1 h. During the final step, the SWCNTs were treated in HNO_3_ at the temperature of 120 °C for 1 h. The defectiveness of the processed CNTs was investigated with a LabRAM HR Raman spectroscope (Horiba, Japan). A spectrum of Raman scattering, excited by 514 nm of laser radiation, was recorded with an accumulation time of 10 s.

**Figure 1:**
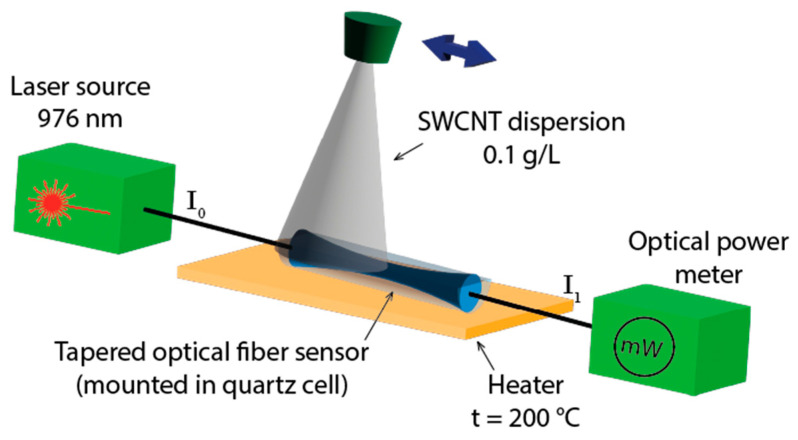
The scheme of the aerosol layer-by-layer deposition.

The concentration of SWCNTs dispersed in dimethylformamide (DMF) was 0.1 g/L. An SWCNT coating was deposited on a tapered section’s surface using the aerosol layer-by-layer method [18]. To form a homogeneous nanotube layer on the sensor surface, the liquid phase of dispersion (DMF) had to be removed. Otherwise, it would lead to the further formation of a large drop in dispersion that would result in the removal of the SWCNT already deposited on the surface. To minimize this effect, the sensor was heated up to 200 °C. The nozzle movement along the sensor axis provided variability in the incidence angle of the nanotube spray, changing with the position of the nozzle, which led to a homogeneous deposited layer. SWCNTs were adhered to the fiber surface using Van der Waals forces; therefore, the utilized method did not require a binder in the content of deposited dispersion. Since the SWCNTs absorbed the light propagating through the optical fiber core, the thickness of the coating was controlled by measuring the reduction of infrared laser radiation to 976 nm during the coating process (Figure 1). The deposition process was stopped when the laser radiation was reduced by up to 60% of the initial power and, as a result, 220 layers were deposited. The tapered fiber sensor was investigated using a scanning electron microscope (SEM) Helios NanoLab 650 (FEI Ltd., Hillsboro, OR, USA).

### 2.3. Experimental Setup

**Figure 2:**
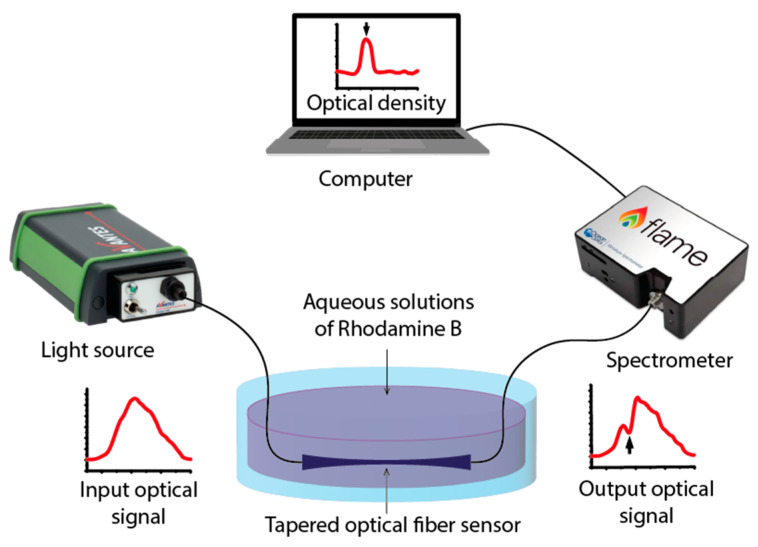
The scheme of the experimental setup.

The experimental setup was comprised of a light source AvaLight-HAL (Avantes, Apeldoorn, The Netherlands) and a spectrometer FLAME-T-XR1-ES (Ocean Optics, Dunedin, FL, USA). Both ends of the tapered fiber sensor were connected to the setup components with bare fiber adapters (Figure 2). The sensor (the tapered part coated with SWCNTs) was located on the bottom of a liquid cell. The radiation from the light source propagated through the optic fiber core and interacted with surrounding media in a tapered section that impacted its spectral properties. The radiation changes were recorded with the spectrometer.

To study sensitive properties, aqueous solutions of dye Rhodamine B were analyzed with the tapered fiber sensor in the order of concentration, as follows: 2, 4, 8, 16, 32, 16, 8, 4, and 2 mg/L. The exposition time was 5 min. After every dye solution, the liquid cell, and especially the sensor, was washed with water in order to remove any presence of the dye. Additionally, air and pure water were also analyzed as background spectra.

## 3. Results and Discussion

### 3.1. Geometric Properties

**Figure 3:**
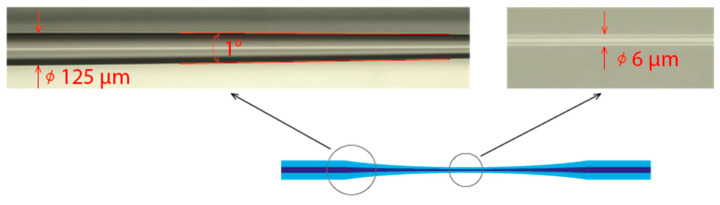
The optical image of the beginning of the taper transition and the taper waist before CNT coating deposition.

The image of the tapered optical fiber sensor (Figure 3) was captured by the 640 × 480 CCD camera of the optical microscope. The diameter of the optical fiber decreased linearly from the initial diameter of 125 µM to 6 µM (at the waist diameter or cladding outer diameter), which was less that the initial core diameter. As a result of the tapering process, the core diameter was reduced from 8.2 µM down to 3.5 µM. The taper angle at the beginning of the transition was about 1° and decreased to the waist, forming a relatively long region with a constant diameter.

A tapered fiber can be considered an adiabatic if most of the optical power remains in the fundamental mode and does not couple with modes of higher orders [24]; hence, the tapered sensor transition region suits the criterion due to the small taper angle. SMF-28 can be considered a multimode fiber at the measurement wavelength. However, evaluating adiabaticity in a multimode fiber taper is a complex task and remains the objective of independent research [25], as does the mode coupling effect during taper production in the presence of high temperatures [26]. However, for the purposes of our study, we have omitted these factors due to the comparative nature of our measurements. Commercial availability dictates the choice of optical fiber. The optical radiation loss through the tapered optical fiber with the described geometry was measured at 1.32 dB.

### 3.2. CNT Characterization

**Figure 4:**
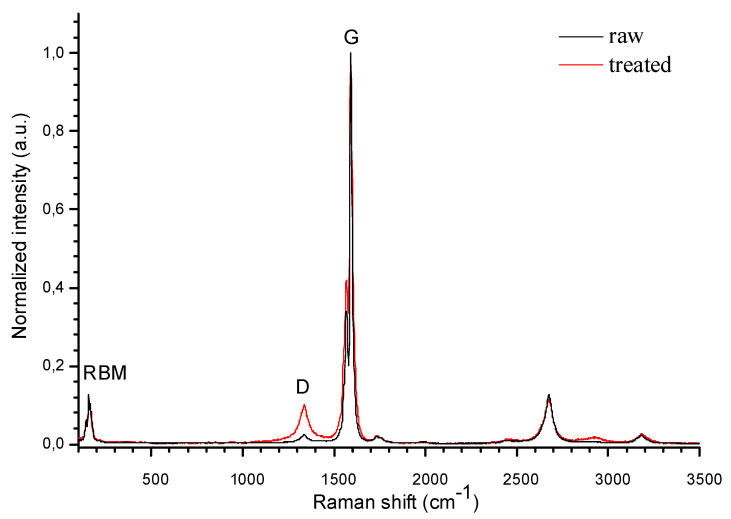
The Raman spectra of the raw nanotubes and the nanotubes treated with SWCNTs.

The Raman spectra of raw and treated CNTs demonstrated the result of the chemical treatment (Figure 4). Both spectra highlighted peaks corresponding to the disorder that occurred in the carbon nanotubes (D-band, 1340 cm^−1^) and the graphitic and well-ordered carbon atoms (G-band, 1590 cm^−1^) [27]. The ratio of the D-band and G-band intensity (I_D_/I_G_) indicated less defective and highly graphitized carbon nanotubes [28]. The I_D_/I_G_ ratio of the raw nanotubes and the treated ones were 0.025 and 0.101, respectively, which indicated a 4-fold change. The value of the I_D_/I_G_ ratio allowed for the evaluation of the crystallite size, L_a_, which characterized the length of the defect-free CNT sections [29]:

L_a_(nm) = (2.4 × 10^−10^)∙λ4laser∙(I_D_∕I_G_)^−1^, (1)

where λ_laser_ is the excitation laser wavelength (nm) used in the Raman spectroscopy.

The crystallite size, L_a_, of the raw carbon nanotubes was 670 nm and was reduced to 166 nm (treated nanotubes) because of the multistage liquid-phase treatment. The L_a_ reduction indicated an increase in the number of defects per unit length, which could improve the adsorption of chemicals on the CNT surface. Additionally, on the treated CNTs’ spectrum there were peaks in the radial breathing mode (RBM) region at 140 cm^−1^ and 160 cm^−1^, which was unique to single-walled carbon nanotubes. These low-frequency peaks allowed for the evaluation of the diameters, which were approximately 1.75 nm and 1.55 nm [30].

**Figure 5:**
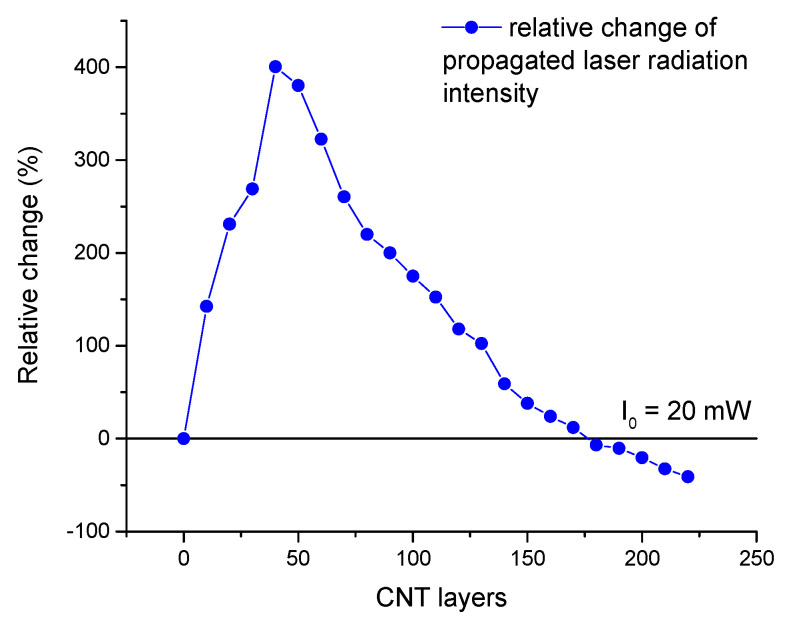
Relative change of laser radiation intensity during the CNT deposition process.

During the CNT deposition process, the laser radiation, with an initial power of 20 mW, changed in a range from 100 mW (+400%) to 12 mW (−40%), as shown in Figure 5. As the first 50 layers were applied, the intensity of the control radiation increased due to the strong solvent DMF cleaning the surface of the sensor [31]. As a result, the deposition of the following layers decreased the control radiation intensity since carbon nanotubes started forming a coating due to high adhesion to the clean surface of the sensor. After 220 cycles of deposition, the control radiation was reduced by up to 60% of the initial power, which was considered to be optimal for the CNTs coating.

**Figure 6:**
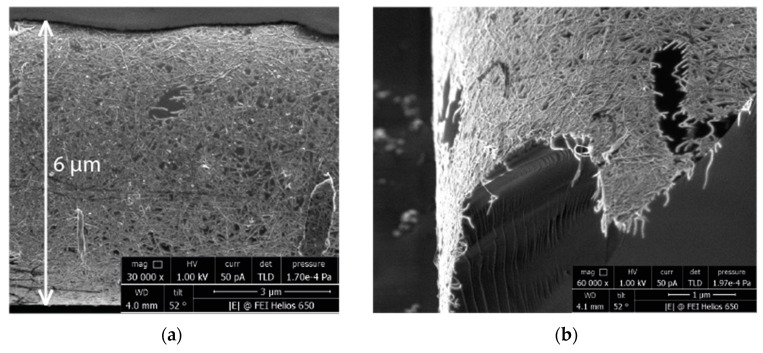
SEM images of SWCNTs thin film coating the tapered sensor waist: plan view (**a**) and cross-section (**b**).

The morphology of the SWCNTs thin film coating the tapered sensor waist was observed using SEM (Figure 6). Nanotubes, deposed by the layer-by-layer method, formed a high-density structure with a chaotic orientation of individual carbon nanotubes and bundles formed by Van der Waals forces. The deposition using the aerosol method resulted in the lack of full coverage of the substrate by the formed SWCNT film [32]. The diameter of the taper waist was 6 µM, which was the same as that measured by optical microscopy. It indicated a low thickness of the SWCNT coating. The SEM image of the coated sensor’s tapered section that was cracked mechanically (Figure 6b) confirmed that the thickness was relatively low (approximately 100 nm).

### 3.3. Optical Transmittance Spectra Performance

The sensitivity performance of the tapered optical fiber sensor was determined as the ratio of the transmitted light intensity through the sensor immersed in water and in an aqueous dye solution (I_water_/I_dye_). Figure 8a shows the I_water_/I_dye_ spectra of Rhodamine B solution at concentrations of 2, 4, 8, 16, and 32 mg/L before and after CNT coating. The spectra of the sensor without CNTs, regardless of the dye concentration, had the same absorption peak at the wavelength of 556 nm, which relates to the results obtained by a commercial spectrometer [33]. However, the spectra of the sensor coated with CNTs showed the Rhodamine B absorbance peak drift for around 8 nm (up to 564 nm) in the solutions with concentrations of 2, 4, and 8 mg/L, and for 11 nm (up to 567 nm), in the solutions with concentrations of 16 and 32 mg/L. The wavelength drift was related to a new dye–SWCNT complex with a different electronic structure that formed upon complexation.

**Figure 7:**
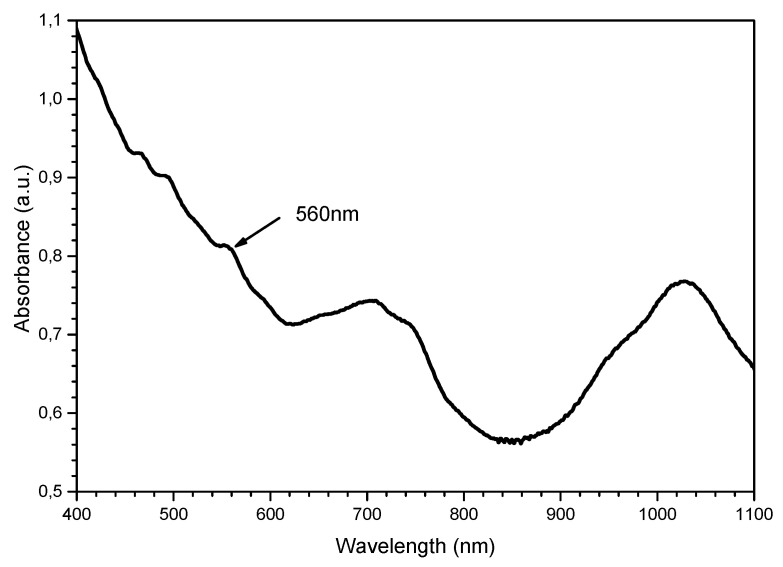
The absorption spectrum of the SWCNT film.

To investigate this fact, the SWCNTs film was deposited on a glass substrate using the aerosol layer-by-layer method (Section 2.2). Then, the absorption spectrum in the VIS-NIR range of the film was recorded (Figure 7). The absorption peak at the wavelength of 560 nm was related to E_33_ optical transitions in semiconducting SWCNTs [34]. As a result, the interaction of dye molecules and nanotubes on the sensor surface led to this significant shift of the dye absorption maximum. Moreover, the parameters of the same Rhodamine B absorption shift in the presence of SWCNTs depend on the carbon nanotubes’ production methods, such as high-pressure pyrolysis (HiPCO) and the Co/Mo catalyst process (CoMoCAT) [35].

**Figure 8:**
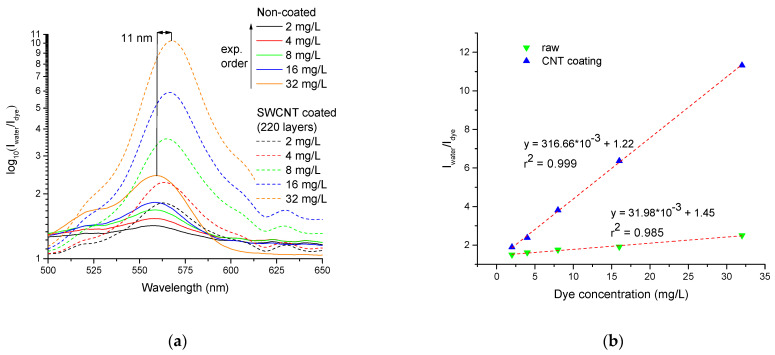
I_water_/I_dye_ spectra of the Rhodamine B solution with concentrations of 2–32 mg/L (solid curves, before CNT deposition; dashed curves, after SWCNTs deposition); (**a**) and I_water_/I_dye_ vs concentration dependences (**b**).

The calibration lines of the sensors were determined as the linear regression of I_water_/I_dye_ vs concentration dependences, calculated using the least squares method (Figure 8b). The sensitivity of the sensor coated with SWCNTs was 317 (mg/L)^−1^ dye concentration and had a slope linearity of 99.9%, while the uncoated sensor sensitivity was 32 (mg/L)^−1^ with a slope linearity of 98.5%. Consequently, the sensor sensitivity was increased by a factor of 10 with the use of SWCNTs, indicating a significant improvement in sensitivity. The sensitivity boost effect of pure SWCNTs demonstrated better performance than SWCNT-polyethylene oxide composite coating [36] and showed an 8× increase in sensitivity.

**Figure 9:**
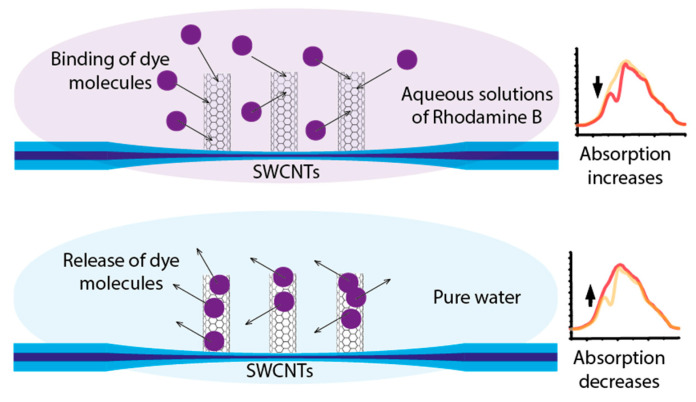
The mechanism of Rhodamine B molecules’ interaction with SWCNTs.

Since the sensing mechanism of the sensor is evanescent wave spectroscopy, the sensitivity depends on the absorption intensity of the analyzed media surrounding the tapered section of the sensor [37]. The evanescent wave propagating through the sensor can only interact with a small volume of dye solution due to the low penetration depth [38,39]. As a result, the absorption intensity of the uncoated sensor is relatively low (Figure 8a, solid curves). In contrast, the sensor coated with SWCNT showed a higher absorption rate for the same dye concentration (Figure 8a, dashed curves). As was described before, Rhodamine B molecules bind to the carbon nanotube surface, which leads to an increase in dye molecule concentration in evanescent wave interaction (Figure 9). The nitric acid treatment allows for the formation of the insoluble SWCNT layer in water [40], which prevents the nanotubes’ removal during experiments with dye solution. Although the treatment also forms OH- and C=O groups [41], the concentration of OH- groups significantly decreases under heating, down to 200 °C during the deposition process (Section 2.2). Finally, C=O groups, principally present on the SWCNT surface, have a chemical affinity for NH_2_ groups of Rhodamine B. It indicates that Rhodamine B molecules bind to nanotube surfaces through physical adsorption, which results in changes in coating optical properties.

The Rhodamine B binding process could continue until an equilibrium state is reached between the concentrations in the dye aqueous solution and on the SWCNT surface. The higher concentration of the analyzed dye surrounding the tapered section of the coated sensor results in higher absorption and, consequently, higher sensitivity. In contrast, SWCNTs with absorbed molecules release dye in pure water and prevent the accumulation effect before the next measurement. It is probable that the sensitivity could be further improved by increasing the number of defects on the SWCNTs surface, which would lead to better binding with Rhodamine B molecules.

**Figure 10:**
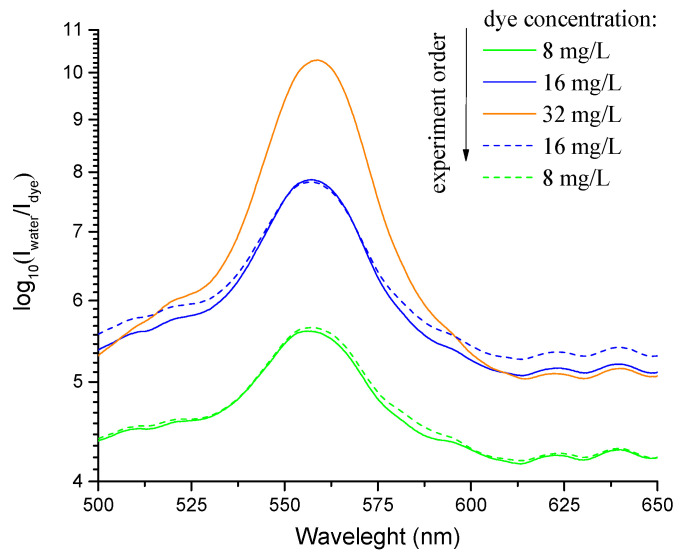
I_water_/I_due_ spectra of the Rhodamine B solution with concentrations analyzed as follows: 8, 16, 32, 16, and 8 mg/L (solid lines, increasing dye concentration; dashed lines, decreasing dye concentration).

The repeatability test was performed in the order shown in Figure 10. In this case, repeatability was concerned with the ability of a tapered optical fiber sensor to produce the same response to the same concentration when the tests were repeated multiple times. The parameter determined the ability to be applied in non-laboratory measurement systems since, in nature, the investigating parameter (concentration) could change chaotically up and down. The test demonstrated the high repeatability of the sensor coated with CNTs during dye solution analysis regardless of the previously analyzed dye concentration value: even the high concentration of 32 mg/L did not result in a significant change in the concentration–optical value dependence. The demonstrated repeatability indicated that the washing process described in 2.3 led to the full removal of Rhodamine B molecules from the surface of the carbon nanotubes coating the tapered optical fiber sensor. The interaction with pure water led to the release of dye molecules (Figure 9). Consequently, in every measurement cycle, it was the actual Rhodamine B concentration in the analyzed media that affected the final absorption intensity, which was used to evaluate the concentration value.

## 4. Conclusions

In summary, the tapered optical fiber sensor with a CNT-nanostructured coating capable of measuring the concentration of Rhodamine B in an aqueous solution has been successfully designed and investigated. A thin, functional coating of CNTs was deposited on the tapered section’s surface using the aerosol layer-by-layer method. The sensor coated with SWCNTs demonstrated significantly higher sensitivity compared with the non-coated sensor in the range of 2–32 mg/L of the Rhodamine B concentration in an aqueous solution. The experimental results showed that the sensitivity was increased 10 times from 32 (mg/L)^−1^ for non-coated sensors up to 317 (mg/L)^−1^ after CNT coating deposition. Moreover, the developed sensor demonstrated high repeatability, which allows for the evaluation of concentration regardless of the previously analyzed dye concentration. Although the sensitivity and repeatability of the utilized evanescent-wave spectroscopy technique are high, the selectivity is limited on the absorption band wavelength of the analyzing materials. This is problematic in the case of the concentration analysis of chemicals with specific absorption bands of similar wavelengths. In summary, the dye sensing performance of the tapered optical fiber sensor coated with CNT points out its potential for the ecological monitoring of the water environment.

## Data Availability

Correspondence and requests for materials should be addressed to Aleksandr A. Polokhin.
